# Ray Marching Aspheric Surfaces: Robust Ray Intersection Calculation for Design of Optical Sensors †

**DOI:** 10.3390/s26144624

**Published:** 2026-07-21

**Authors:** Vadim Sanzharov, Sergey Ershov, Vladimir Frolov, Vladimir Galaktionov, Alexey Voloboy

**Affiliations:** 1 Institute for Artificial Intelligence, Lomonosov Moscow State University, Leninskie Gory 1-52, 119991 Moscow, Russia; vadim.sanzharov@graphics.cs.msu.ru (V.S.); vladimir.frolov@graphics.cs.msu.ru (V.F.); 2 Keldysh Institute of Applied Mathematics RAS, Miusskaya Sq. 4, 125047 Moscow, Russia; ersh@gin.keldysh.ru (S.E.); vlgal@gin.keldysh.ru (V.G.)

**Keywords:** optical sensors, optics, lens systems, ray tracing, aspherical surfaces

## Abstract

When designing lens systems for optical sensors, a fundamental operation is finding the intersection of a ray and the lens surface. We propose a robust method for finding the intersection of a ray with highly aspherical rotationally symmetric surfaces. Our method provides reliable results for ray incident at large angles relative to the optical axis and successfully calculates intersection points for a significantly larger number of rays than existing methods, improving the reliability of optical modeling and design. The core idea of our method is to calculate an initial approximation of the intersection point for the Newton method using a ray marching procedure with a step size estimated form of analytical derivatives on the surface function. The proposed method does not require maintaining additional complex data structures and is suitable for use in the iterative optimization of optical systems where the surface geometry frequently changes.

## 1. Introduction

Aspherical lens systems are widely used in modern sensors and cameras, including built-in smartphone cameras, due to their high image sharpness, compact size and light weight. They enable high image resolution and accuracy, essential for medical imaging devices, laser scanners, micro- and telescopes. Computer-aided design of such systems requires highly accurate ray path modeling. Accurate and efficient ray tracing serves as important computational foundation for the design and analysis of existing and emerging optical systems.

Emerging class of optical sensors utilize metasurface designs [1,2] that exploit optical symmetry. Such metasurface designs can be combined with conventional refractive and free-form aspheric elements. For example, the authors of [3] integrate a polarization-multiplexed metasurface with freeform optics in a joint optimization loop to create a compact AR display with three independent focal planes. Meanwhile, the work [4] proposes an active retinal projection display in which pixel-level collimators of microlenses are co-optimized with aspheric relay elements. Thus, in such a new optical sensor design, the ability to accurately trace rays through the conventional elements that can serve as a substrate and/or relay optics, remains a direct prerequisite for accurate system-level simulation and optimization.

The core of this process is the problem of calculating the precise intersection point of a ray with an optical surface. For non-spherical surfaces, such as high-order polynomial aspheres and freeform representations, the intersection point is typically found by solving a nonlinear equation derived from the sagittal representation of the surface. The standard numerical approach is the Newton iterative method due to its quadratic convergence near the solution.

However, the convergence of the Newton method critically depends on providing a sufficiently accurate initial guess. In the context of ray tracing, the choice of this initial guess is a key factor affecting the overall performance of the algorithm. An incorrectly chosen initial guess can lead to divergent iterations, convergence to a non-physical root, or excessive computational overhead. Therefore, a reliable initial guess selection strategy is important for two main reasons: first, to ensure reliable convergence over a wide range of ray directions and surface geometries, preventing failures in the ray-tracing pipeline; and second, to improve computational efficiency, since a near-optimal initial guess minimizes the number of Newton iterations required to achieve a prescribed tolerance, which directly impacts simulation speed for systems requiring the analysis of millions of rays.

Let us consider a ray with origin *o* and direction *v*

(1) p(t)=o+v·t

and the problem of finding its intersection point with rotationally symmetric surfaces used in optical modeling and design that have the form $z=f(r^{2})$, such as even polynomial aspheres, Q-type polynomials, cubic splines, and others. Since the intersection point lies on both the surface $z=f(r^{2})$ and the ray p(t), it can be found by solving the following equation for *t*:

(2) $$z_{surf}-z_{ray}=f\left({\left(o_{x}+v_{x}\cdot t\right)}^{2}+{\left(o_{y}+v_{y}\cdot t\right)}^{2}\right)-\left(o_{z}+v_{z}\cdot t\right)=0$$

All roots of Equation (2) are intersection points of which only the first intersection point (i.e., the one closest to the ray origin *o*) is often needed. For Newton’s method to produce a solution corresponding to the first intersection point, it is necessary to specify an initial guess sufficiently close to this point.

To find an initial guess for the first intersection, we propose a method similar to the family of ray marching algorithms. Ray marching is typically applied to implicitly defined surface functions. In the most basic form, the ray marching algorithm steps along the ray path at equidistant intervals and samples the surface function. The intersection point is assumed to be located in the interval where the sign changes. Robustness of the intersection search is ensured by using a sufficiently small step size, which, however, leads to significant computational cost.

Adaptive step selection can alleviate expenses. Various techniques have been proposed to compute lower bound on the distance to the surface for certain classes of functions. The most widely used is the Lipschitz bound, which underpins Sphere Tracing [5] and Segment Tracing [6]. Alternative approaches include Chebyshev-based estimates [7] and bounds derived from Harnack inequalities [8]. Moreover, it was shown in [9] that Lipschitz bounds can be interpreted as forward inclusion functions—bounds that are exact at the beginning of the ray interval and grow monotonically with the ray parameter. Quadratic inclusion functions provide a more accurate approximation of curved fields, allowing the use of larger, but still safe, step sizes. In this work, we integrate and adapt elements of these methods for explicitly defined, twice-differentiable, rotationally symmetric surfaces expressed as $z=f(r^{2})$ where *r* is the radial coordinate.

The contribution of this paper is a robust deterministic algorithm for ray intersection with aforementioned surface types that reduces the number of missed intersections to less than 0.001% while maintaining memory and computational efficiency. The algorithm provides a reliable initial guess for the Newton method by marching along the ray with an adaptively chosen step size, derived from analytically precomputed bounds on the first and second derivatives of the surface sag function over a set of radial subintervals. The safe step is a strict lower bound on the distance to the first intersection within the current interval, guaranteed by the bracketing inequalities developed in Section 3.1. The method requires no auxiliary acceleration structures, no heuristic initial-guess selection, and no mesh or point-grid representations of the surface.

The rest of the paper is organized as follows: Section 2 surveys the work on ray–surface intersection; Section 3 derives the step-size bounds and describes the full algorithm; Section 4 evaluates the method against established baselines on both a challenging synthetic benchmark and a realistic sequential ray tracing workload; Section 5 concludes and outlines directions for future work.

## 2. Related Work

In optical system modeling, there are several approaches to calculating the initial guess for Newton’s method. Many methods linearize or simplify the surface locally to obtain a first approximation. The simplest approach is to use a plane perpendicular to the optical axis. This can be a plane tangent to the optical surface or a plane passing through its center. The ray equation is substituted into the plane equation, and the resulting solution is used as an initial estimate for Newton’s method.

Similarly, the intersection with a sphere instead of a plane can be used as an initial approximation. For aspherical surfaces defined as a base conic (e.g., a sphere or paraboloid) plus polynomial terms, the intersection with the base sphere can be used as an initial guess [10]. The intersection of the ray with the base sphere can be found analytically and yields a point closer to the actual surface that the plane approximation.

However, using the initial approximation obtained in this way does not guarantee the convergence of Newton’s method. For surfaces with complex shapes and/or for rays propagating at large angles, the plane or sphere approximation may be insufficient. If the surface has a bend or significant curvature, the intersection point with the tangent plane may lie outside the physical aperture, causing Newton’s method to converge to an incorrect solution. Typically, such approximations work if the surface is sufficiently close to flat or spherical in the region of interest.

In [11], the authors estimate the initial approximation, ray step, and intersection search interval using two analytical surfaces that bound the optical surface on both sides. Spheres or planes are proposed as the bounding surfaces. When calculating ray intersections with lens surfaces, the authors prefer the bisection method with an initial search interval defined by the bounding surfaces. The main drawback of the bisection method is that it requires several times more iterations than Newton’s method to achieve the high level of accuracy required in optical calculations.

In general, analytical approximations using a plane or sphere lack robustness for complex, highly aspherical shapes, but are simple to implement and require minimal computational effort. Therefore, they are widely used in ray tracing of optical systems, including recent works such as [12,13,14].

Some approaches try to substitute the intersection calculation with Newton’s method by converting surface into the patches [15] or a fine triangulated mesh [16], for which an analytical solution for ray intersection exists. However, fine meshes require significant memory and computational resources. Therefore, other approaches use coarse-sampled grids to localize the intersection and then apply Newton’s method.

In [17], triangulation is used to find intersections for freeform surfaces modeled by B-splines. The authors exploit the properties of B-splines to potentially speed up the process of finding intersection points between rays and triangles. However, the performance and efficiency of the proposed approach were not investigated and are left for future research.

In [18], a mesh-based approach is used for freeform NURBS surfaces. To reduce the dependence on a fine grid, the authors replace the surface parameters (u,v) with a transformed pair (ξ,η) that lies in the interval [0,1], even if the estimates for (u,v) fall outside this interval. Thus, Newton’s method will converge to the desired solution even for initial values ($u_{0}$,$v_{0}$) selected from a sufficiently coarse grid. One drawback of this approach is that the intersection computation procedure is generally more complex for these methods, since both the forward and inverse transformations must be implemented.

To improve the efficiency of intersection search using polygonal mesh representations of surfaces, acceleration structures (such as a bounding volume hierarchy, BVH) can be used. In [19], this approach is proposed for aspherical surfaces, and in [20], its application is extended to freeform surfaces. Preliminary intersection with triangulated meshes provides a good initial guess, which accelerates convergence and allows for the handling of problematic rays, such as tangent (or nearly tangent) to the surface. BVH can also be used for NURBS and spline surfaces, where a typical strategy is to flatten or refine the control mesh until each patch is nearly flat, then build an axis-aligned BVH over those patches [21]. However, in optical system design problems, where the target surface is continually changing during the optimization process, using this approach can be challenging, since the triangulated geometry and acceleration structure must also be continually updated.

Instead of triangles, a simple grid of points on the target aspheric surface can be used. The authors of [22] propose placing a set of reference points on the target surface within the aperture and finding the closest point (in terms of Euclidean distance) among them for each incoming ray. The found point is then projected perpendicularly onto the ray and used as an initial guess for Newton’s method. Essentially, this approach emits a ray near a known surface point. This ensures that the initial guess is close to the surface and improves convergence for highly aspheric or freeform surfaces, avoiding convergence to incorrect roots that can occur with methods using plane approximation. The disadvantage is the additional cost of storing reference points and searching for the closest point. Furthermore, in optical system design problems, reference points must be recalculated each time the surface is iteratively modified.

Robust ray intersection algorithms are studied not only for optical modeling and design, but also as a fundamental challenge in computational geometry and computer graphics. In these areas, parametric surfaces are typically considered. In [23], interval analysis is applied to guarantee both the existence of a unique ray intersection with parametric surface and the convergence of Newton iteration from any starting point within a suitably defined parameter region. The main drawback of this approach is its high computational cost.

Paper [24] presents a hybrid ray–Bézier surface intersection algorithm that combines the Newton method with Bézier clipping and exploits the coherence of neighboring rays. While this approach is effective for coherent primary rays in computer graphics, it is less suitable for optical design, where rays are not coherent across scan lines. Furthermore, it is limited to Bernstein basis surfaces, a necessary condition for underlying clipping and projection operations.

The authors of [25] present a local iterative ray–surface intersection method that employs the second-order Taylor expansion of the surface along its intersection curve with the plane containing the ray. The approach remains stable when the Jacobian is ill-conditioned or singular. However, it still depends on the initial guess, has no convergence guarantees, and can fail in complex cases with more than two intersections.

In [26], the ray–surface intersection problem is formulated as an initial value problem governed by first-order ODEs along the surface curve. A line segment is constructed from an arbitrary point on the surface to the ray and projected onto the surface; the resulting curve satisfies geometric ODEs, and its numerical integration directly yields an intersection point. Although the method reliably finds intersections from any starting point, it does not guarantee the closest intersection point, which is often necessary in optical modeling, and is poorly suited to cases with multiple ray intersections.

Overall, simple and fast methods based on analytical approximation do not provide a good enough initial approximation to ensure convergence. Methods based on triangular, patch or point grids provide an initial approximation close to the solution, but require significant computational costs and memory resources to build and store new representations, limiting their applicability.

## 3. Proposed Method

Overall, the proposed method for acquisition of intersection points consists of the following steps:

1. Obtaining an initial coarse approximation of the intersection point that will serve as the starting point for ray marching, using a simple method based on bounding planes.
2. Refining the intersection point approximation using iterative marching along the ray with adaptive steps.
3. Calculating the intersection point with the required precision using Newton’s method, starting from the approximation found in the previous step.

### 3.1. Step Size Estimation

The lower bound for the intersection distance is derived from the bounds of the surface function. We begin by rewriting Equation (2) as follows:

(3) $$f\left(r_{0}^{2}+a\cdot t^{2}+b\cdot t\right)-\left(o_{z}+v_{z}\cdot t\right)=0$$

where

(4) $$\begin{array}{cc} & r_{0}^{2}\equiv o_{x}^{2}+o_{y}^{2}\\ \; & a\equiv 1-v_{z}^{2}\\ \; & b\equiv 2\cdot (o_{x}\cdot v_{x}+o_{y}\cdot v_{y}) \end{array}$$

Next, we divide the possible values of $r^{2}$ within aperture into equal radial intervals $\left[R_{i}^{2},R_{i+1}^{2}\right]$. One option is to use the mean value theorem to obtain linear bounds of the surface function values in each interval from the minimum and maximum values of the first derivative $f^{\prime }\left(r^{2}\right)$:

(5) $$f\left(r_{0}^{2}\right)+\left(r^{2}-r_{0}^{2}\right)\cdot f_{min,i}^{\prime }\leq f\left(r^{2}\right)\leq f\left(r_{0}^{2}\right)+\left(r^{2}-r_{0}^{2}\right)\cdot f_{max,i}^{\prime }\;\;\;\;\;for\;\;\;\;r^{2}\in \left[R_{i}^{2},R_{i+1}^{2}\right]$$

The inequality (5) in combination with Equation (3) produces two equations that bound the value of the root *t*:

(6) $$\begin{array}{c} f\left(r_{0}^{2}\right)+\left(a\cdot t^{2}+b\cdot t\right)\cdot f_{min,i}^{\prime }-\left(z_{0}+v_{z}\cdot t\right)=0\\ f\left(r_{0}^{2}\right)+\left(a\cdot t^{2}+b\cdot t\right)\cdot f_{max,i}^{\prime }-\left(z_{0}+v_{z}\cdot t\right)=0 \end{array}$$

We call this approach “ray marching with linear bounds”.

Then we calculate the bounds of the second derivative $f^{\prime \prime }$ within each interval $\left[R_{i}^{2},R_{i+1}^{2}\right]$:

(7) $$f_{min,i}^{\prime \prime }\leq f^{\prime \prime }\left(r^{2}\right)\leq f_{max,i}^{\prime \prime }\;\;\;\;\;for\;\;\;\;r^{2}\in \left[R_{i}^{2},R_{i+1}^{2}\right]$$

These bounds are computed over the full interval with extrema of $f^{\prime \prime }$ at or near the boundary captured within the interval.

Then, for the interval containing $r_{0}^{2}\in \left[R_{i}^{2},R_{i+1}^{2}\right]$, a second-order Taylor expansion can be used, similar to [9], to express the bounds of the surface function $f(r_{0}^{2}+a\cdot t^{2}+b\cdot t)$:

(8) $$\begin{array}{cc} f\left(r_{0}^{2}+a\cdot t^{2}+b\cdot t\right) & \geq f\left(r_{0}^{2}\right)+\left(a\cdot t^{2}+b\cdot t\right)\cdot f^{\prime }\left(r_{0}^{2}\right)+\frac{1}{2}{\left(a\cdot t^{2}+b\cdot t\right)}^{2}\cdot f_{min,i}^{\prime \prime }\\ f\left(r_{0}^{2}+a\cdot t^{2}+b\cdot t\right) & \leq f\left(r_{0}^{2}\right)+\left(a\cdot t^{2}+b\cdot t\right)\cdot f^{\prime }\left(r_{0}^{2}\right)+\frac{1}{2}{\left(a\cdot t^{2}+b\cdot t\right)}^{2}\cdot f_{max,i}^{\prime \prime } \end{array}$$

These estimates hold for $t\leq t_{c}$ where $t_{c}$ is the constraint defined as the distance *t* where $r_{ray}^{2}\left(t\right)$ leaves the interval $\left[R_{i}^{2},R_{i+1}^{2}\right]$:

(9) $$t_{c}=\left\{\begin{array}{cc} -\frac{b}{2\cdot a}-\frac{\sqrt{b^{2}+4\cdot a\cdot \left(R_{i}^{2}-r_{0}^{2}\right)}}{2\cdot a}, & b\leq 0\\ -\frac{b}{2\cdot a}+\frac{\sqrt{b^{2}+4\cdot a\cdot \left(R_{i+1}^{2}-r_{0}^{2}\right)}}{2\cdot a}, & b\geq 0 \end{array}\right.$$

Once the ray exits the interval, the algorithm moves to the next interval and recalculates the bounds.

**Figure 1:**
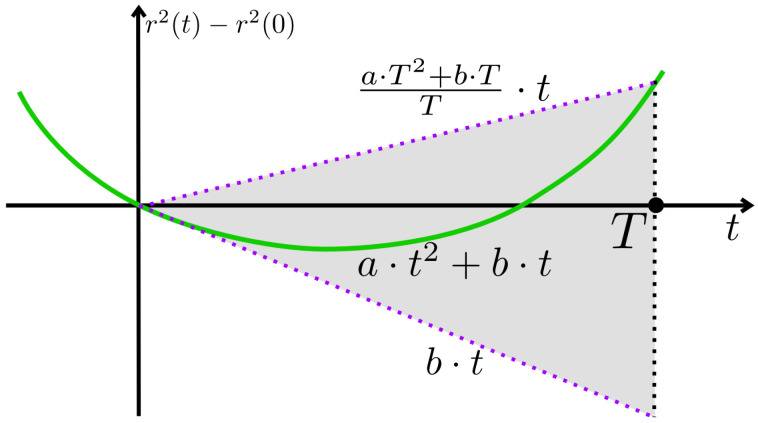
Parabola bracketing with a chord and a tangent.

The equations on the right side of the inequalities (8) are fourth-degree equations in *t* making their direct solution computationally expensive. To avoid solving these fourth-degree equations, the term $\left(a\cdot t^{2}+b\cdot t\right)$ can be bracketed. For 0≤t≤T the parabola $a\cdot t^{2}+b\cdot t$ lies between its tangent line b·t and the chord connecting the points at t=0 and t=T (see Figure 1). Taking $T=t_{c}$, where $t_{c}$ is the largest value for which Equation (8) holds, we obtain:

(10) $$\begin{array}{c} b\cdot t\leq \left(a\cdot t^{2}+b\cdot t\right)\leq \left(b+a\cdot t_{c}\right)\cdot t,\;b\leq 0\\ b\cdot t\geq \left(a\cdot t^{2}+b\cdot t\right)\geq \left(b+a\cdot t_{c}\right)\cdot t,\;b\geq 0 \end{array}$$

Notice that if b≥0 then the bounds are swapped. The square of $at^{2}+bt$ is then bounded by:

(11) $$\beta_{\min}\cdot t^{2}\leq {\left(a\cdot t^{2}+b\cdot t\right)}^{2}\leq \beta_{\max}\cdot t^{2}$$

where

(12) $$\begin{array}{c} \beta_{\min}\equiv \left\{\begin{array}{cc} \min\left(b^{2},{\left(b+a\cdot t_{c}\right)}^{2}\right), & b\;\;\mathrm{and}\;\;(b+a\cdot t_{c})\;\;\mathrm{have}\;\;\mathrm{the}\;\;\mathrm{same}\;\;\mathrm{sign}\\ 0, & b\;\;\mathrm{and}\;\;(b+a\cdot t_{c})\;\;\mathrm{have}\;\;\mathrm{different}\;\;\mathrm{sign} \end{array}\right.\\ \beta_{\max}\equiv \max\left(b^{2},{\left(b+a\cdot t_{c}\right)}^{2}\right) \end{array}$$

Denoting

(13) $$\begin{array}{cc} & \gamma_{\min,i}\equiv \left\{\begin{array}{cc} \frac{\beta_{\max}\cdot f_{min,i}^{\prime \prime }}{2}, & f_{min,i}^{\prime \prime }\leq 0\\ \frac{\beta_{\min}\cdot f_{min,i}^{\prime \prime }}{2}, & f_{min,i}^{\prime \prime }\geq 0 \end{array}\right.\\ \; & \gamma_{\max,i}\equiv \left\{\begin{array}{cc} \frac{\beta_{\min}\cdot f_{max,i}^{\prime \prime }}{2}, & f_{max,i}^{\prime \prime }\leq 0\\ \frac{\beta_{\max}\cdot f_{max,i}^{\prime \prime }}{2}, & f_{max,i}^{\prime \prime }\geq 0 \end{array}\right. \end{array}$$

and combining Equation (13) with the inequalities (8), we finally get the following inequalities expressing the bounds of the surface function:

(14) $$\begin{array}{c} f\left(r_{0}^{2}+a\cdot t^{2}+b\cdot t\right)\geq f\left(r_{0}^{2}\right)+b\cdot f^{\prime }\left(r_{0}^{2}\right)\cdot t+\left(a\cdot f^{\prime }\left(r_{0}^{2}\right)+\gamma_{\min,i}\right)\cdot t^{2}\\ f\left(r_{0}^{2}+a\cdot t^{2}+b\cdot t\right)\leq f\left(r_{0}^{2}\right)+b\cdot f^{\prime }\left(r_{0}^{2}\right)\cdot t+\left(a\cdot f^{\prime }\left(r_{0}^{2}\right)+\gamma_{\max,i}\right)\cdot t^{2} \end{array}$$

The root *t* of Equation (3) is then bracketed by the positive roots of the two quadratic equations:

(15) $$\begin{array}{cc} \left(f\left(r_{0}^{2}\right)-z_{0}\right)+\left(b\cdot f^{\prime }\left(r_{0}^{2}\right)-v_{z}\right)\cdot t+\left(a\cdot f^{\prime }\left(r_{0}^{2}\right)+\gamma_{\min,i}\right)\cdot t^{2}= & 0\\ \left(f\left(r_{0}^{2}\right)-z_{0}\right)+\left(b\cdot f^{\prime }\left(r_{0}^{2}\right)-v_{z}\right)\cdot t+\left(a\cdot f^{\prime }\left(r_{0}^{2}\right)+\gamma_{\max,i}\right)\cdot t^{2}= & 0 \end{array}$$

Since Equation (15) is satisfied only inside the interval $r^{2}\in \left[R_{i}^{2},R_{i+1}^{2}\right]$, the following procedure must be used to estimate the “safe” step along the ray:

1. Calculate the constraint value $t_{c}$ such that $r_{0}^{2}+a\cdot t_{c}^{2}+b\cdot t_{c}$ leaves $\left[R_{i}^{2},R_{i+1}^{2}\right]$ using Equation (9).
2. For each of the two equations in (15), calculate the roots and take the smallest positive value (in the absence of positive root, we adopt +∞). As a result, we obtain two values: $t_{1}$ (from the first equation) and $t_{2}$ (from the second equation).
3. The ray cannot intersect the surface at $t\leq \min(t_{1},t_{2},t_{c})$ and it is safe to take a step to the point defined by $min(t_{1},t_{2},t_{c})$

It is guaranteed that the ray will not cross the surface before this safe step as the bounding quadratics (Equation (15)) lie entirely below (or above) the target equation by construction, so the estimated step cannot overshoot the first intersection.

When a ray is nearly tangent to the surface, the value of *b* approaches zero. In this case, the parabola is bounded almost entirely by the chord bracket with constraint $t_{c}$ likely becoming the primary limit on the step size. This results in the shorter steps and slower convergence but does not violate the guarantee; the process remains correct, though potentially slower.

Determining a safe step size for ray marching with linear bounds follows the same procedure, except that the second stage instead calculates the roots of Equation (6).

**Figure 2:**
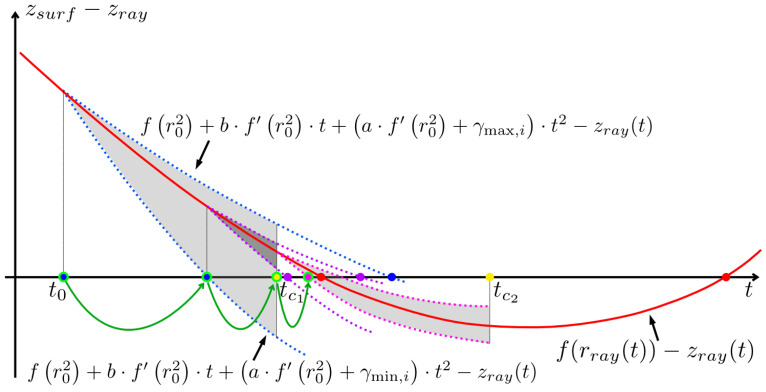
Ray marching algorithm with quadratic bounds. The red curve shows the target equation, and the red dot indicates its root (the intersection of the ray with the surface); the dashed lines show the root bracketing at different iterations of the algorithm; the dots outlined in green show successive intersection distance estimates.

Figure 2 shows three sequential steps of ray marching using quadratic bounds. The gray curved triangles show the bracketing region, enclosed by the bounds obtained using the inequalities (8) and the constraint value (Equation (9), shown as yellow dots $t_{c1}$ and $t_{c2}$) for the interval $\left[R_{i}^{2},R_{i+1}^{2}\right]$ containing the current value of $r_{0}^{2}$. An algorithmic description of one step of ray marching with quadratic bounds is presented in Algorithm 1.

Estimating step size assumes that the surface sag function $f(r^{2})$ must be twice differentiable on $\left[0,R_{max}^{2}\right]$. This is satisfied by all surface families considered in the paper.
**Algorithm 1** One ray marching step using quadratic bounds.**Require:**
      Function $\mathrm{RAYSTEP}(r^{2},a,b,\Delta F)$ returns Δt

      Input parameters: $r^{2}$—radial coordinate, a,b—parameters computed from Equation (4),
      ΔF—left side of Equation (3) at t=0
**Ensure:** Step along the ray Δt, such that there is no intersection between current distance
      value $t_{0}$ and $t_{0}+\Delta t$

1:   $f_{max,i}^{\prime \prime },f_{min,i}^{\prime \prime },R_{i},R_{i+1}=\mathrm{DERIVATIVEBOUNDS}\left(r^{2},b\right)$        ▹ As in Equation (7)

2:   $t_{c}=\mathrm{CONSTRAINT}\left(a,b,r^{2},R_{i},R_{i+1}\right)$     ▹ Compute constraint from Equation (9)

3:   $\gamma_{min},\gamma_{max}\leftarrow \mathrm{GAMMA}\left(a,b,t_{c},f_{max,i}^{\prime \prime },f_{min,i}^{\prime \prime }\right)$            ▹ From Equation (13)

4:   $t_{1}\leftarrow \mathrm{SOLVEQUADRATIC}\left(A=a\cdot f^{\prime }+\gamma_{min},B=b\cdot f^{\prime }-v_{z},C=\Delta F\right)$▹ Returns smallest positive root of $A\cdot x^{2}+B\cdot x+C=0$

5:   $t_{2}\leftarrow \mathrm{SOLVEQUADRATIC}\left(A=a\cdot f^{\prime }+\gamma_{max},B=b\cdot f^{\prime }-v_{z},C=\Delta F\right)$

6:   $\Delta t\leftarrow \mathrm{MINIMUM}t_{c},t_{1},t_{2})$

7:   **return** 
Δt


### 3.2. Ray Marching Algorithm

A ray marching algorithm is presented in Algorithm 2.

As a result of the derivation, the described ray marching adaptive step algorithm provides the lower bound estimate for the ray free path length. To obtain an iterative algorithm, the estimated safe step is used to update the ray origin:

(16) $$o_{new}=o+v\cdot min\left(t_{1},t_{2},t_{c}\right)$$

and the same computations are repeated.

Algorithm does not assume that the ray remains in the same single interval. The current value of $r_{0}^{2}$ is updated at each iteration (Algorithm 2, line 3) and inside each invocation of the RayStep, corresponding interval is determined with the bounds freshly recomputed.

Since SolveQuadratic always takes the smallest positive root, i.e., Δt>0, the marching always advances forward from the current ray origin. Since the ray origin is located before the surface and the algorithm never steps backward, it is guaranteed that Algorithm 2 stops at the first sign change, i.e., the first root encountered in the forward direction. This exactly corresponds to the first physical intersection.
**Algorithm 2** Ray marching smooth radially-symmetric surface.**Require:**     Function $\mathrm{APPROXINTERSECTION}(o,d,\epsilon_{1},\epsilon_{2})$ returns $t_{0},valid$     Maximum valid radial coordinate $R_{\max}^{2}$, maximum number of iterations iter_max**Ensure:** Approximate distance $t_{0}$ such that $\mathrm{SURFACE}\left({\left(o_{x}\right)}^{2}+{\left(o_{y}\right)}^{2}\right)-\left(o_{z}+t_{0}\cdot d_{z}\right)\leq \epsilon_{2}$1:   $a\leftarrow {\left(d_{x}\right)}^{2}+{\left(d_{y}\right)}^{2}$, $\hat{o}\leftarrow o$, $t_{0}\leftarrow 0$, $\Delta F_{prev}=+inf$, $\xi \leftarrow 10^{-5}$, $\sigma \leftarrow 10^{-10}$2:   **for** iter←0 to iter_max **do**3:        $b\leftarrow 2\cdot ({\hat{o}}_{x}\cdot d_{x}+{\hat{o}}_{y}\cdot d_{y})$, $r^{2}\leftarrow {\hat{o}}_{x}\cdot {\hat{o}}_{x}+{\hat{o}}_{y}\cdot {\hat{o}}_{y}$4:        $f,f^{\prime }\leftarrow \mathrm{SURFACEANDDERIVATIVE}\left(r^{2}\right)$             ▹ Surface function5:        $\Delta F=f-{\hat{o}}_{z}$6:        **if** $|\Delta F|<\epsilon_{2}$ **then**               ▹ Run trial Newton iteration7:             $dt\leftarrow b\cdot f^{\prime }-d_{z}$8:             δ←−ΔF/dt9:             **if** $|\delta |<\epsilon_{1}^{2}$ and $t_{0}+\delta >\xi$ **then**10:                 $t_{0}\leftarrow t_{0}+\delta$11:                 **return** $t_{0},valid\leftarrow True$12:             **end if**13:        **end if**14:        **if** iter>0 and $sign\left(\Delta F_{prev}\right)=-sign\left(\Delta F\right)$ **then**15:             **return** $t_{0},valid\leftarrow True$             ▹ previous intersection16:        **end if**17:        $\Delta F_{prev}\leftarrow \Delta F$18:        $\Delta t\leftarrow \mathrm{RAYSTEP}(r^{2},a,b,\Delta F)$               ▹ See Algorithm 119:        **if** Δt<σ and $r^{2}>R_{max}^{2}-\sigma$ and b>0 **then**  ▹ Reached $R_{max}^{2}$, no intersection20:             **return** −1,valid←False21:        **end if**22:        $t_{0}\leftarrow t_{0}+\Delta t$23:        $\hat{o}\leftarrow o+t_{0}\cdot d$24:   **end for**25:   **return** −1,valid←False

In successive marching steps, the lower bound on the ray–surface distance estimate increases monotonically, while the step size decreases accordingly. Throughout the process, the estimate remains strictly below the true intersection point, so it is necessary to introduce a termination criterion to stop the marching. One convenient option is to monitor the ray-surface distance (i.e., the left-hand side of Equation (3)) and terminate when this value falls below a prescribed tolerance ϵ (line 6 of Algorithm 2).

After the marching stage, Newton’s method is employed to refine the intersection distance *t* to the desired precision. However, it is difficult to guarantee the convergence of Newton iterations from a given starting point. If ϵ is chosen too small, the marching phase may require an excessive number of steps; if it is too large, Newton’s method may diverge or converge to an unintended root.

To address this issue, after the main stopping condition is satisfied, we perform a trial Newton method step (lines 7–13 of Algorithm 2). This continues until the distance between the ray and the surface drops below $\epsilon^{2}$, indicating quadratic convergence, or until the maximal number of iterations is reached. We then permanently switch to the Newton method.

Another situation that needs to be considered is when the true intersection lies outside the physical aperture of the surface. In such situations, the marching algorithm may never satisfy the previously defined stopping criteria unless an explicit iteration limit is imposed. This occurs because the radial intervals $\left[R_{i}^{2},R_{i+1}^{2}\right]$ used for the squared radius $r^{2}$ are defined only within the aperture. If the actual intersection lies outside this region, the marching process is limited to the first or last interval, since the $t_{c}$ constraint prevents the ray from going beyond the allowed range. Although this situation does not lead to incorrect results, it wastes computational resources. Therefore, it is necessary to check whether the current intersection estimate corresponds to a value of $r^{2}$ equal to or greater than the maximum allowed value. When this condition is detected, it can be concluded that the ray does not intersect the surface inside the aperture, and tracing for this ray can be stopped (line 16 of Algorithm 2).

### 3.3. Coarse Intersection Approximation

The ray origin *o* can be located at an arbitrary distance from the optical surface. If the origin is located far away, then marching would be inefficient. Therefore, it is beneficial to first obtain a coarse estimate of the intersection point and use this estimate as the starting point for the subsequent ray-marching stage. The simplest such estimate is the ray’s intersection with a pair of bounding planes that enclose the surface in the *z* axial direction.

To construct these bounding planes the minimum ($z_{min}$) and maximum ($z_{max}$) values of the surface sag function are first determined. This is achieved by sampling the surface at a reasonable number of radial positions (we used 101), with the squared radius $r^{2}$ varying from 0 (the optical axis) to the aperture edge, and evaluating both the sag and its derivative at each sampling point. If the derivative changes sign between two consecutive samples at $r_{i}^{2}$ and $r_{i+1}^{2}$, the local extremum—and hence a candidate for $z_{min}$ or $z_{max}$—lies in that interval. In such cases, the surface can be locally approximated by a cubic polynomial Equation (17) over the interval, which provides a more accurate estimate of the extremal sag value.

(17) $$\begin{array}{cc} & p\left(x\right)=a_{0}+a_{1}\cdot x+a_{2}\cdot x^{2}+a_{3}\cdot x^{3}\\ \; & x=r_{i+1}^{2}-r_{i}^{2} \end{array}$$

The coefficients of the polynomial are derived from the system:

(18) $$\left\{\begin{array}{cc} p(0) & =a_{0}=f_{i}\\ p^{\prime }\left(0\right) & =a_{1}=f_{i}^{\prime }\\ p(x) & =a_{0}+a_{1}\cdot x+a_{2}\cdot x^{2}+a_{3}\cdot x^{3}=f_{i}\\ p^{\prime }\left(x\right) & =a_{1}+2\cdot a_{2}\cdot x+3\cdot a_{3}\cdot x^{2}=f_{i}^{\prime } \end{array}\right.$$

If $p^{\prime }\left(x\right)=0$, then the quadratic equation has roots giving candidate values of *x*, where the cubic may have a local extremum. If the root lies in the interval $\left[0,r_{i+1}^{2}-r_{i}^{2}\right]$, then the surface function *f* at this coordinate should be used to update the global values $z_{min}$ and $z_{max}$ accordingly.

A coarse intersection point is then determined as the intersection of the ray with the plane defined by $z_{min}$ or $z_{max}$, depending on which is closer to the ray origin.

The proposed ray marching algorithm requires evaluating the surface sag function together with its first and second derivatives. However, in some cases, the sag may be undefined or numerically unstable for certain radial coordinates $r^{2}$. Therefore, before starting the marching process, it is necessary to ensure that the squared radius associated with the ray origin is within the admissible range $r^{2}\leq r_{max}^{2}$, where $r_{max}^{2}$ is usually determined by the aperture *A* ($r_{max}^{2}=A^{2}$). Depending on the particular surface formulation, additional constraints may be required. For example, polynomial aspherical surfaces contain a conic term that imposes additional constraints on the feasible range of $r^{2}$ (Equation (19)).

(19) $$\frac{c\cdot r^{2}}{1+\sqrt{1-\left(1+\kappa \right)\cdot c^{2}\cdot r^{2}}}$$

where *c*—surface curvature and κ—conic.

The term under the square root in Equation (19) remains valid only when κ>−1.0 and $r^{2}\geq \frac{1}{\left(1+\kappa \right)\cdot c^{2}}$. Therefore, the admissible radial coordinate must satisfy

(20) $$r^{2}\leq r_{max}^{2}=min\left\{\frac{1}{\left(1+\kappa \right)\cdot c^{2}},A\right\}$$

If the coarse intersection point calculated from the bounding plane approximation is outside this interval, it should be replaced by a point on the ray for which $r^{2}\left(t\right)=r_{max}^{2}$. This point can be found by solving the quadratic equation

(21) $$\left(d_{x}^{2}+d_{y}^{2}\right)\cdot t^{2}+2\cdot \left(o_{x}\cdot d_{x}+o_{y}\cdot d_{y}\right)\cdot t+o_{x}\cdot o_{y}-r_{max}^{2}=0$$

and choosing the smallest positive root. If the quadratic equation has no real solutions, the ray does not intersect the surface within the allowed aperture.

## 4. Results and Discussion

The proposed intersection search algorithm was evaluated in two contexts: (1) sequential ray tracing through a complete lens system and (2) a specially designed synthetic benchmark intended to test the robustness of intersection search.

**Figure 3:**
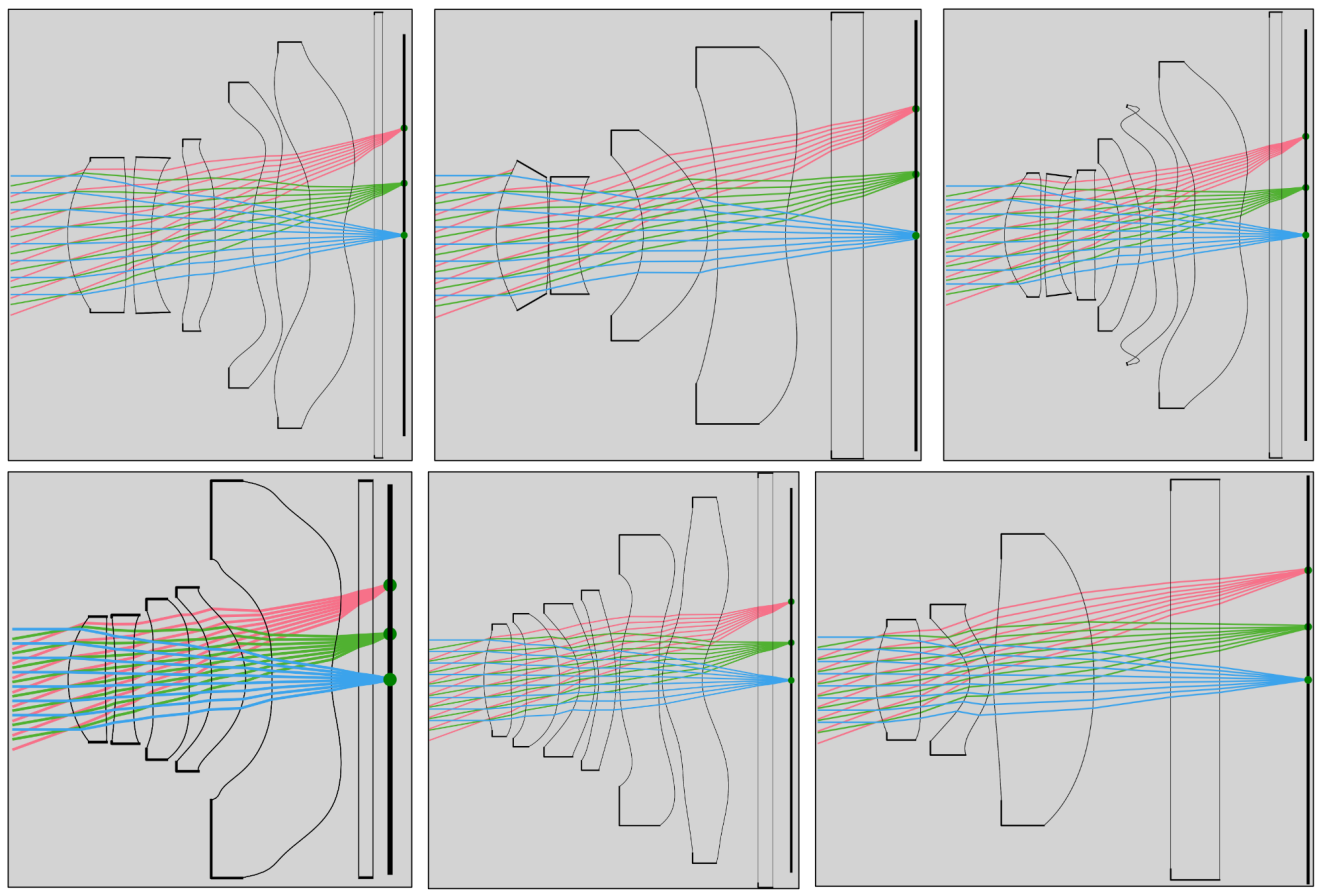
Representative surface sag profiles of the designs from the test dataset. Double-curved “W” surfaces typically present the greatest challenges for simple methods. In the case of the synthetic benchmark each surface of the design is tested for intersections separately. Different ray colors mark different input beams and are not explicitly related to a wavelength.

For these tests we used a dataset of 978 aspherical surfaces extracted from mobile phone lens designs. These surfaces were generated automatically by an optimization pipeline that employs automatic differentiation and gradient descent techniques; representative designs are shown in Figure 3. The different colors of the rays if the images just indicate the different ray beams.

Our method was compared with two established approaches: (i) a coarse approximation based on intersection with bounding planes, which is fast, simple and widely adopted; (ii) the reference point method [22], which requires only a set of sampled surface points and does not rely on expensive data structures; (iii) hybrid approach that Newton’s method with bisection fallback in two variants: in the first variant initial guess and bracketing are obtained from bounding planes and in the second variant uses fixed step ray marching.

All compared methods use identical Newton refinement with the same tolerance $\epsilon_{1}$, and hence achieve equivalent final residuals when converging to the correct root.

To ensure objective comparison, the reference point algorithm was re-implemented in C++ in the same programming environment as the other methods (gcc compiler version 13.03; the original Python version 3.12 and PyTorch 2.8 were ported). Its only configurable parameter is the density of the reference point grid on the surface.

The parameters of Algorithm 2 used in the experiments are as follows. The Newton’s method tolerance is $\epsilon_{1}=10^{-6}\;\mathrm{mm}=10^{-9}\;m$, the marching tolerance is $\epsilon_{2}=10^{-3}$ and the safeguard is $\sigma =10^{-10}$. The Sigma is needed to understand that the steps are already very small, and we are approaching the border of the maximum radial coordinate, which means that there is no intersection within the limits of the aperture. The maximum number of the Newton’s method iterations—24, the maximum number of the ray marching iterations—100, and the maximum number of the hybrid method iterations (bisection/Newton)—512. The maximum allowable radial coordinates $R_{max}^{2}$ were calculated for each surface according to Section 3.3. The radial coordinates were subdivided into four intervals for the ablation study, and the derivative bounds were calculated by uniformly sampling 20 points in each interval.

Roughly speaking, $\epsilon_{1}$ and $\epsilon_{2}$ determine the stop criterion for the ray marching procedure, i.e., when to stop the ray marching iterations and pass the approximate root to the final Newton’s method. This criterion is two-level: at the first level, it compares the discrepancy |ΔF| in the intersection equation with the $\epsilon_{2}$, line 6 in Algorithm 2; if it is small enough, then we move to the second level, performing single trial Newton’s iteration and checking whether the improved root $t_{0}+\Delta t$ differs from the bracketing value $t_{0}$ by only at most $\epsilon_{1}$. If the second level check fails with, ray marching continues.

### 4.1. Synthetic Test

**Figure 4:**
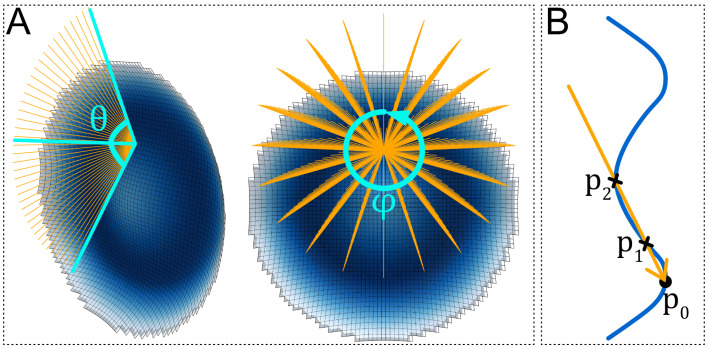
(**A**) Generated rays from a single point example. (**B**) Ray constructed from some point on a surface $p_{0}$ can intersect surface in other locations $p_{1}$ and $p_{2}$.

The synthetic benchmark was constructed by sampling a set of points $\{p_{0}^{i}\},i=1,\ldots ,N$ (for testing we used N=101) on each surface and generating rays that terminate at those points. Each ray was defined by two angular parameters, ϕ and θ, and a distance from its origin to a target point on the surface. The angle θ was sampled with 21 uniformly spaced values ranging from 0° to 60° for two directions symmetrical about the optical axis; the angle ϕ was sampled uniformly from 0° to 2π (42 values). The distance from the origin to the surface was also sampled uniformly with 21 values ranging from 0.01 to three aperture radii. All possible combinations of surface point, ϕ, θ, and distance yielded a total of 1,870,722 test rays. A subset of these rays for a single surface point is illustrated in Figure 4A.

Because many of the generated rays intersect the surface more than once (Figure 4B), the point $p_{0}^{i}$ used to create a given ray is not necessarily the first intersection point. Therefore, the intersection search was evaluated using the following procedure:

1. Failure to find an intersection point. The ray is considered a missed intersection.
2. An intersection was found but not at the point $p_{0}^{i}$. The ray origin is moved to the found intersection point, and the search is repeated. This iteration is performed up to 100 times.
3. The intersection coincides with $p_{0}^{i}$. The ray is considered successfully traced.

This benchmark represents a substantially more challenging intersection search problem than a typical sequential ray tracing through a lens assembly. Nevertheless, such difficult rays do arise in practice; for example, in stray-light analysis or in optical systems with mirrors. The purpose of the proposed test is to check whether the algorithm is able to reliably find any intersections.

The results (Table 1, Missed intersections) demonstrate that the proposed method achieves an extremely low miss rate, outperforming competing approaches by several orders of magnitude. In the worst case, the bounding plane and reference point methods fail to locate up to 5–6% of intersections (approximately 100,000 rays), while our algorithm maintains a near-perfect success rate, typically with one missed intersection ray per million rays. The hybrid approach combined with bounding planes provides only a modest improvement over the plain Newton method with the same initial guessing strategy. This is likely due to insufficiently tight bracketing provided by bounding planes which can be crucial in the proposed benchmark. When the initial guess and bracketing calculation are performed using a fixed step of marching along the ray until a sign change, the improvement is more noticeable. However, the results are highly dependent on the chosen fixed step size. We present results for only one step size, but they illustrate that the combination of the number of missed intersections and performance is inferior to the proposed solution. This highlights the advantage of the proposed method which constructs the brackets adaptively.

For the reference point method [22], increasing the grid resolution beyond the 15 points reported in Table 1 produces only a slight increase in the intersection success rate, while significantly degrading performance due to the quadratic increase in computational cost with respect to resolution.

### 4.2. Sequential Ray Tracing Test

The sequential ray tracing experiment was designed to evaluate the algorithm’s performance in a realistic optical design workflow. For each lens system we traced a bundle of 512 × 512 rays with varying incidence angles. The total execution time was recorded, including an initial coarse approximation and subsequent refinement using Newton’s method. All tests were run on an AMD Ryzen 9 5950X CPU (16 cores/32 threads), and the ray tracing loop was parallelized across rays using the OpenMP library. To compile C++ code we used gcc 13.03 under Ubuntu Linux 24.04.1.

The computational overhead of our method is modest: on average, it is only 1.5–1.6 times slower than a simple bounding plane approach (see Table 1). Memory consumption is also minimal, requiring storage of only the extrema $f_{max}^{\prime \prime },f_{min}^{\prime \prime }$ for each radial-squared subdivision interval. The improved initial guess generated by the proposed algorithms also reduces the number of iterations required by Newton’s method. This trade-off improves overall performance, as the additional time spent on ray marching is offset by the faster convergence (fewer iterations) of Newton’s method.

The results in Table 1 were obtained using four quadratically bounded subdivision intervals for ray marching and four hierarchical levels of linearly bounded subdivisions with 4, 16, 32, and 64 intervals, respectively.

**Figure 5:**
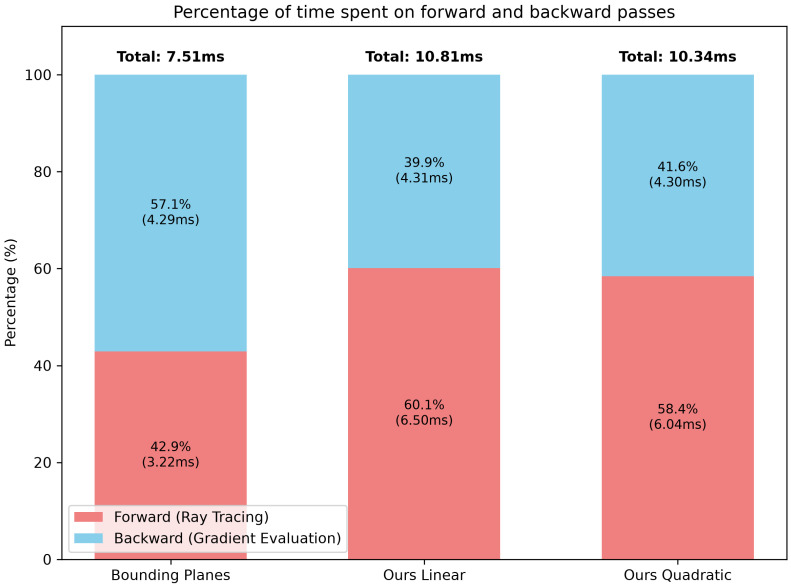
Comparison for average execution time per surface for forward and backward pass for surfaces from Figure 3. The ray tracing bundle is 512×512 rays.

Within the overall optimization pipeline of the optical system, the proposed method introduces a slight increase in computational cost per iteration. The gradients were calculated using analytically derivation, and on average take from 40% to 60% of the optimization step time. The corresponding measurements are presented in Figure 5 for the surfaces from Figure 3.

### 4.3. Ablation Study

A comprehensive ablation study was conducted to validate the necessity of each step in our method. Numerical results in Table 2 are provided for ray marching with quadratic bounds. Similar behavior and conclusions hold for the linearly bounded variant because the steps examined belong to the outer iterative marching loop (Algorithm 2) and Newton refinement, which are identical in both cases. Specifically, the ablation study was conducted by removing one or more of the following parts of the algorithm:

- A—a single trial Newton iteration;
- B—solution refinement with iterative Newton’s method;
- C—coarse approximation by bounding plane;
- D—stopping condition for missing rays.

When the tolerances $\epsilon_{1}$ and $\epsilon_{2}$ are set sufficiently low, the intersection distance can often be obtained solely using the ray marching algorithm (Algorithm 2), omitting both the single trial Newton iteration (A) and the subsequent iterative refinement by Newton’s method (B). Although this reduces the execution time marginally, it leads to a noticeable increase in the number of missed intersections on the synthetic benchmark.

Excluding only the trial Newton step (A), that is incorporated into the marching termination criterion, also results in a significant increase in the fraction of missed intersections.

Removing the initial coarse intersection estimate (C) obtained from the bounding plane approximation forces the algorithm to perform many more marching steps, that, as expected, degrades the overall ray tracing performance.

Without the stopping condition for missing rays (D), the performance of the proposed algorithm degrades significantly, increasing the execution time by more than 10 times.

The choice of interval subdivision for the squared radius region $\left[R_{i}^{2},R_{i+1}^{2}\right]$ noticeably affects the behavior of algorithm. A finer subdivision tightens the bounds in Equation (8), yielding a more accurate bracket around the true intersection. However, it also affects the value of the constraint $t_{c}$, leading to the reduced size of the valid region; when $t_{c}$ becomes very small, the bounds $f_{min}^{\prime \prime }$ and $f_{max}^{\prime \prime }$ can no longer be used, and the safe marching step is limited to only $t_{c}$, which may be inefficient. Therefore, depending on the ray direction and surface geometry, both coarse or fine subdivision can produce larger permissible steps.

Our empirical study (Table 3) shows that for a ray marching with quadratic bounds, the best compromise is achieved by subdivision into four intervals. It minimizes the missed intersection rate without sacrificing performance.

In the case of linear bounds, a multi-level subdivision strategy is advantageous. For example, computing candidate steps using 4, 8, 32 and 64 intervals and then choosing the largest feasible step among them yields better results.

## 5. Conclusions and Future Work

In this paper, we propose a robust method of ray intersection with highly aspherical, rotationally symmetric surfaces. The method addresses a well-known challenge with the Newton-based intersection solvers: sensitivity to the quality of the initial guess, which leads to missed or incorrect intersections for rays incident at large angles or on surfaces with complex sag profiles. By marching along the ray with a step size derived from analytically bounded first and second derivatives of the sag function, the algorithm guarantees that no intersection is missed within each radial subinterval. This deterministic property distinguishes our method from heuristic initialization strategies.

Experimental evaluation on a dataset of aspherical surfaces from mobile phone lens designs demonstrates that the proposed method reduces the number of the missed intersections by several orders of magnitude compared to baseline methods based on bounding planes and reference points, achieving a rate of only 0.001% in the synthetic benchmark, while incurring the runtime by only 1.5–1.6 times compared to a simple bounding plane approach. The improved initial guess also reduces the number of required Newton iterations, partially offsetting the cost of the marching phase. Memory consumption remains minimal—two scalars per radial subinterval—making the method well suited for iterative optimization of optical systems, where the surface geometry changes at each design step and expensive precomputed data structures must be re-created. All this makes it suitable for use in the optimization and design of optical sensors (particularly for mobile devices, laser scanners, micro- and endoscopes).

The method is currently formulated for rotationally symmetric surfaces of the form $z=f(r^{2})$. Extending the underlying ideas—precomputed derivative bounds over a subdivided domain and marching with quadratic inclusions—to XY-polynomial and Zernike freeform surfaces, off-axis conics, and NURBS representations remains an important direction for future work. Such extensions will require replacing the one-dimensional radial marching with a two-dimensional search in parameter space and developing similar safe step estimates for more general surface equations.

## Figures and Tables

**Table 1:** Comparison of our ray marching algorithms with competitors. The hybrid method is Newton + bisectio. All values are averaged over the surface. The ray tracing bundle is 512×512 rays. The number of Newton method iterations is given for a ray bundle at 40°.

Method	Newton or Bisection Iterations	Missed Intersections		Average Time, ms		
		Average %	Maximum %	Rays at 0°	Rays at 20°	Rays at 40°
Bounding planes	18.52	0.24	6.12	3.01	3.48	3.70
Reference points, N=15	21.01	0.19	5.44	21.99	23.29	23.58
Hybrid, bounding planes	20.65	0.22	4.94	9.91	9.95	10.31
Hybrid, fixed march, 32 steps	12.31	0.13	1.74	22.89	23.71	25.6
Ours, linear bounds	4.57	0.0002	0.07	4.45	6.41	6.73
Ours, quadratic bounds	4.57	$6\times 10^{-6}$	0.002	4.30	6.09	6.1

**Table 2:** Ablation study. The proposed method in full is A + B + C + D. Average tracing time is given for 512×512 rays bundle at 40°.

Variant	Average Missed Rays, %	Maximum Missed Rays, %	Average Marching Iterations	Average Tracing Time, ms
C + D	0.26	7.29	10.2	5.99
B + C + D	0.08	0.44	13.2	5.62
A + C + D	0.079	0.51	29.30	5.61
A + B + D	0.0006	0.13	24.08	8.54
A + B + C	0.0006	0.13	100.70	64.89
A + B + C + D	$6\times 10^{-6}$	0.002	10.19	6.11

**Table 3:** Study of subdivision intervals Average tracing time is given for 512×512 rays bundle at 40°.

Subdivision Intervals	Average Missed Intersections, %	Maximum Missed Intersections, %	Average Marching Iterations	Average Tracing Time, ms
2	$5\times 10^{-4}$	0.26	9.29	5.06
4	$6\times 10^{-6}$	0.002	10.36	6.12
8	$1\times 10^{-5}$	0.004	13.83	9.09
16	$1\times 10^{-5}$	0.002	21.67	15.09
32	$2\times 10^{-5}$	0.004	38.53	27.81

## Data Availability

Data underlying the results presented in this paper are not publicly available at this time but may be obtained from the authors upon reasonable request.
